# How well do large language model-based chatbots perform in oral and maxillofacial radiology?

**DOI:** 10.1093/dmfr/twae021

**Published:** 2024-06-07

**Authors:** Hui Jeong, Sang-Sun Han, Youngjae Yu, Saejin Kim, Kug Jin Jeon

**Affiliations:** Department of Oral and Maxillofacial Radiology, Yonsei University College of Dentistry, Seoul 03722, Republic of Korea; Department of Oral and Maxillofacial Radiology, Yonsei University College of Dentistry, Seoul 03722, Republic of Korea; Institute for Innovation in Digital Healthcare, Yonsei University, Seoul 03722, Republic of Korea; Oral Science Research Center, College of Dentistry, Yonsei University, Seoul 03722, Republic of Korea; Department of Artificial Intelligence, Yonsei University College of Computing, Seoul 03722, Republic of Korea; Department of Artificial Intelligence, Yonsei University College of Computing, Seoul 03722, Republic of Korea; Department of Oral and Maxillofacial Radiology, Yonsei University College of Dentistry, Seoul 03722, Republic of Korea; Institute for Innovation in Digital Healthcare, Yonsei University, Seoul 03722, Republic of Korea

**Keywords:** large language model, artificial intelligence, chatbot, education, dental, oral and maxillofacial radiology

## Abstract

**Objectives:**

This study evaluated the performance of four large language model (LLM)-based chatbots by comparing their test results with those of dental students on an oral and maxillofacial radiology examination.

**Methods:**

ChatGPT, ChatGPT Plus, Bard, and Bing Chat were tested on 52 questions from regular dental college examinations. These questions were categorized into three educational content areas: basic knowledge, imaging and equipment, and image interpretation. They were also classified as multiple-choice questions (MCQs) and short-answer questions (SAQs). The accuracy rates of the chatbots were compared with the performance of students, and further analysis was conducted based on the educational content and question type.

**Results:**

The students’ overall accuracy rate was 81.2%, while that of the chatbots varied: 50.0% for ChatGPT, 65.4% for ChatGPT Plus, 50.0% for Bard, and 63.5% for Bing Chat. ChatGPT Plus achieved a higher accuracy rate for basic knowledge than the students (93.8% vs. 78.7%). However, all chatbots performed poorly in image interpretation, with accuracy rates below 35.0%. All chatbots scored less than 60.0% on MCQs, but performed better on SAQs.

**Conclusions:**

The performance of chatbots in oral and maxillofacial radiology was unsatisfactory. Further training using specific, relevant data derived solely from reliable sources is required. Additionally, the validity of these chatbots’ responses must be meticulously verified.

## Introduction

The recent development of artificial intelligence (AI) has led to the emergence of large language model (LLM)-based chatbots that are easily accessible to the general public. This accessibility has helped make them one of the most intensely discussed topics across various sectors. The release of OpenAI's ChatGPT in November 2022, an interactive AI chatbot that utilizes GPT-3.5 and GPT-4, marked a significant shift in the landscape of language AI. While it took Facebook 10 months to reach one million users, ChatGPT achieved this milestone in just five days. In response, major tech companies entered the fray; Microsoft launched Bing Chat in February 2023 and Google followed with Bard in March 2023.

Because LLM-based chatbots are used for writing papers, school assignments, and reports, journal submission guidelines have been issued for the use of AI,^1^ and educators have begun to recognize the need for a paradigm shift. Active debates have occurred regarding potential restrictions on use and the correct ways to use AI. Many corporations have started offering ChatGPT-based customer service, and both businesses and individuals have developed AI-reliant productivity improvements. Recently, there has been a significant surge of interest in the medical field.^2–4^

ChatGPT has been reported to pass graduate-level and certification exams in fields such as medicine,^5^ law,^6^ and business,^7^ with rapid performance improvements. In the medical domain, performance evaluations were conducted not only for ChatGPT but also for other chatbots across various specialties such as neonatology,^8^ nursing,^9^ neurosurgery,^10^ radiology,^11^^,^^12^ and respiratory medicine.^13^ In particular, ChatGPT’s success in passing the United States Medical Licensing Exam (USMLE) without special training marked a significant milestone in the use of AI chatbots in medicine and garnered global attention in January 2023.^5^ Although reports on the potential of chatbots in dentistry only began to be published towards the end of 2023, ongoing research continues to emerge in general dentistry^14–16^ and in specific fields such as periodontics,^17^ orthodontics,^18^ and oral and maxillofacial surgery.^19–21^ However, to our knowledge, no studies have yet been reported in the field of oral and maxillofacial radiology.

Given the undisclosed scope of chatbots’ training data, variability in performance across both general and specialized domains is anticipated. Differences may also arise among products from various companies. This is the first study to evaluate LLM-based chatbots’ level of performance in comparison to dental students in the field of oral and maxillofacial radiology, and the hypothesis was that the chatbots would perform as well as the students. To assess the influence of educational content and question type on accuracy, we analysed accuracy according to these factors. This research provides insights into the potential of chatbots as reference tools in oral and maxillofacial radiology, and highlights the feasibility of utilizing them within this specific dental field.

## Methods

### Study design and test questions

**Figure 1.**
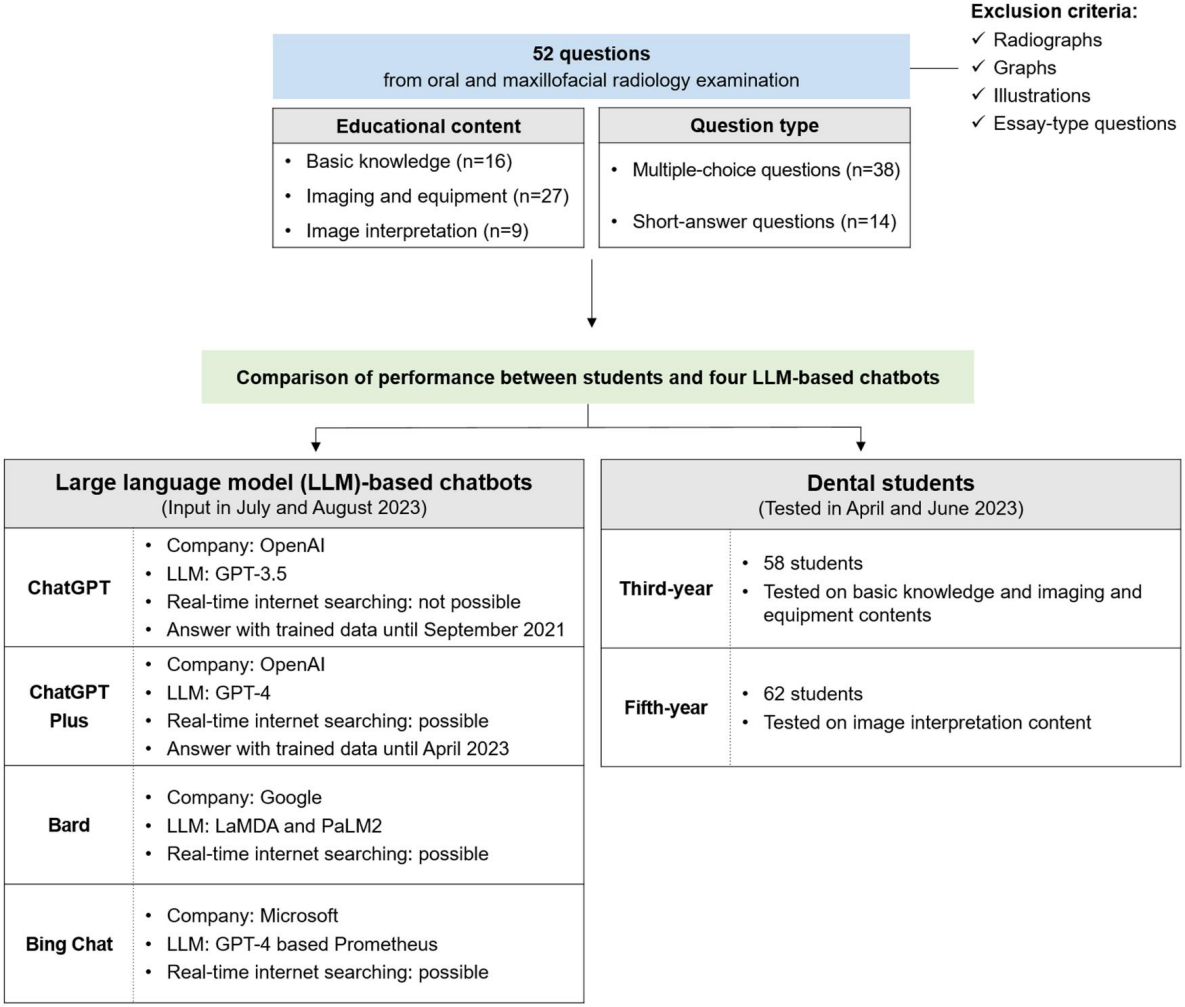
The overall workflow of this study and four LLM-based chatbots.

Figure 1 shows the overall workflow of this study. The study utilized questions and results from the regular oral and maxillofacial radiology examinations conducted at Yonsei University College of Dentistry in April and June 2023. Fifty-two questions were selected, excluding questions containing radiographs, graphs, and illustrations, since images could not be uploaded into the chatbots. Essay-type questions that were difficult to objectively evaluate were also excluded. All questions were prepared in Korean, the native language of Korean dental students, and were presented to the chatbots without any English translation (Supplementary Appendix S1). The questions were divided into three categories based on their educational content: basic knowledge (16 questions), imaging and equipment (27 questions), and image interpretation (nine questions). The questions were also divided into 38 multiple-choice questions (MCQs) and 14 short-answer questions (SAQs) based on question type. The answer format for the selected questions was straightforward: MCQs required the selection of a single correct answer from several options, and SAQs required the use of key conceptual terms (Table 1).

**Table 1.** Question data from an oral and maxillofacial radiology examination, divided by educational content and question type.

Educational content	Question type	Example
Basic knowledge (*n* = 16)	MCQs (*n* = 14)	Choose the most accurate statement about the characteristic that differentiates X-rays from visible light Choose the most accurate statement about factors influencing radiographic density
	SAQs (*n* = 2)	( ) refers to the time it takes for the number of atoms of a radioactive isotope to be reduced by one-half due to radioactive decay ( ) was introduced by the International Commission on Radiological Protection (ICRP) in 1996, where the radiation doses from radiological and nuclear medicine examinations conducted at national or regional medical institutions are surveyed and represented in percentiles, followed by presenting a reference radiation dose. This involves investigating the imaging conditions and radiation doses for diagnostic imaging at medical institutions nationwide, then establishing the reference level at 75th percentile of the distribution of patient exposure doses
Imaging and equipment (*n* = 27)	MCQs (*n* = 16)	Choose the most inaccurate statement about the advantages of panoramic radiography compared to intraoral radiography Choose the most accurate statement about a component that is not necessarily required to generate and utilize cone-beam CT images
	SAQs (*n* = 11)	The main ingredient of the contrast medium used in MR imaging is ( ) The primary goal of infection prevention is to prevent ( ) between patients, and between patients and practitioners
Image interpretation (*n* = 9)	MCQs (*n* = 8)	Choose the most inaccurate statement about endocrine disorders manifesting in the oral and maxillofacial region Choose the most inaccurate statement regarding imaging diagnoses of the temporomandibular joint
	SAQ (*n* = 1)	Among extraoral radiographs, ( ), taken with reduced radiation exposure, is useful for detecting zygomatic bone fractures

Abbreviations: *n* = number of questions; MCQs = multiple-choice questions;  SAQs = short-answer questions.

### Dental students

As part of their curriculum, third-year college students received 32 hours of traditional instruction from an oral and maxillofacial radiologist with 29 years of experience. The lectures covered fundamental topics such as physics, the biological effects of ionizing radiation, and radiation dosimetry and protection. Additionally, the curriculum included discussions on imaging and equipment, including intraoral and extraoral projections, panoramic radiographs, cone-beam CT (CBCT), CT, and MRI. Fifth-year students received 16 hours of traditional instruction from an oral and maxillofacial radiologist with 26 years of experience. These lectures focused on the interpretation of images related to oral and maxillofacial trauma, systemic diseases, temporomandibular joint disorders, and other conditions affecting bone structure.

A total of 120 dental students, comprising 58 third-year students and 62 fifth-year students, took the mid-semester and end-of-semester examinations developed by the radiologist responsible for each course. Neither individual consent from the students nor institutional review board approval was required because grading students’ exams is a standard practice in dental schools.

### LLM-based chatbots and input queries

The questions were entered into four LLM-based chatbots (ChatGPT, ChatGPT Plus, Bard, and Bing Chat) during July and August 2023, with the results analysed from July to September 2023. OpenAI's ChatGPT and ChatGPT Plus, as well as Microsoft's Bing Chat, are based on the GPT-3.5, GPT-4, and GPT-4-based Prometheus models, respectively. Google's Bard is based on the Language Model for Dialogue Applications (LaMDA) and the Pathways Language Model (PaLM). Unlike ChatGPT, all other chatbots also incorporate real-time internet searching.

The questions were entered in Korean after the following prompt engineering: for MCQs that required a single response, the additional instruction to “choose the most accurate/inaccurate statement” was included. All SAQs, whether originally with or without blank spaces, were standardized to a format that included blank spaces (Table 2).

**Table 2.** Example of a short-answer question with the prompt engineered to include a blank space.

	Example
SAQ without blank space	What is the ingredient of contrast medium used in MR imaging?
SAQ with blank space	The main ingredient of the contrast medium used in MR imaging is ( )

Abbreviation: SAQ = short-answer question.

### Performance measurement of students and the four chatbots

To measure the performance of students and four chatbots, instructors from each course independently created test questions and reviewed the answers three times to minimize potential grading errors. For MCQs, answers were simply categorized as correct or incorrect to ensure an unbiased evaluation process. In the case of SAQs, the answers did not take the form of essays nor require the respondent’s inferential skills; therefore, scoring these questions did not require subjective judgment from the evaluators. Answers that deviated from the standard or contained spelling errors were marked incorrect.

To closely replicate the exam conditions that students encounter, a “single-input” strategy was adopted,^22^ where each question was entered into the chatbots only once. The evaluation focused solely on the correctness of the response, without considering the underlying rationale. All test questions were equally scored. This method aimed to ensure objectivity and provide a fair assessment of the chatbots' performance in an environment similar to that of actual exams.

### Data analysis

A descriptive analysis was conducted to compare the overall accuracy rates of dental students and four LLM-based chatbots. Further analysis was carried out to evaluate the results according to the educational content and types of questions.

## Results

Table 3 shows the accuracy rates of dental students compared to four LLM-based chatbots. The overall accuracy rate for the students was 81.2%, while ChatGPT Plus achieved an accuracy rate of 65.4%.

**Table 3.** Accuracy rates (%) for the dental students and four LLM-based chatbots.

		Accuracy rate (%)
Dental students		81.2
	ChatGPT	50.0
	ChatGPT Plus	65.4
Chatbots	Bard	50.0
	Bing Chat	63.5

**Figure 2.**
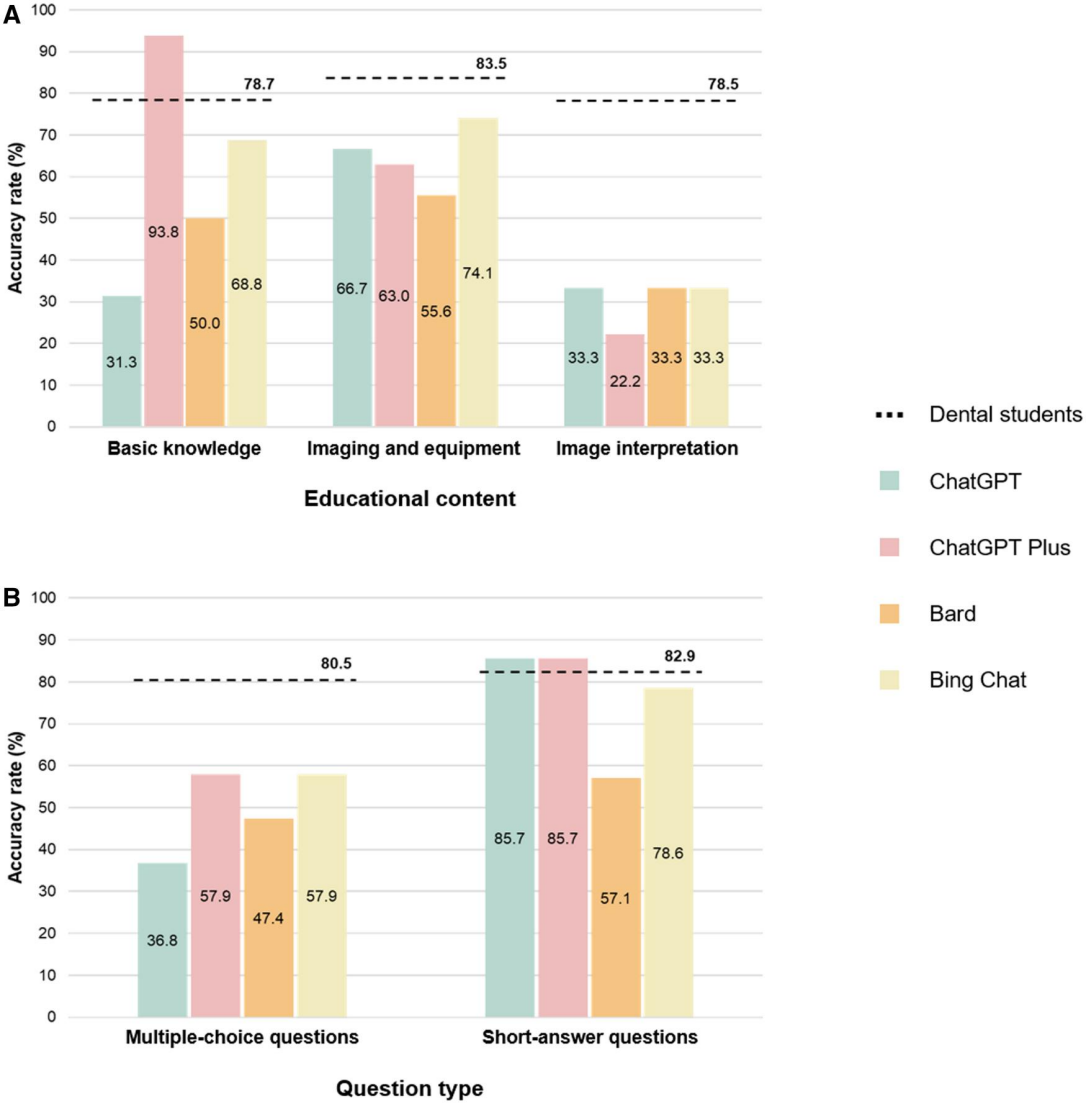
Accuracy rates (%) of four LLM-based chatbots according to educational content (A) and question type (B).

Dental students’ accuracy rates for questions on basic knowledge, imaging and equipment, and image interpretation were 78.7%, 83.5%, and 78.5%, respectively. ChatGPT Plus had an accuracy rate of 93.8% for basic knowledge, and Bing Chat scored 74.1% for imaging and equipment. ChatGPT, Bard, and Bing Chat had accuracy rates of 33.3% for image interpretation (Figure 2A). The dental students had an accuracy rate of 80.5% for MCQs and 82.9% for SAQs. ChatGPT Plus achieved accuracy rates of 57.9% and 85.7% for MCQs and SAQs, respectively (Figure 2B).

## Discussion

Various LLM-based chatbots, including the well-known ChatGPT, are increasingly being utilized in diverse applications despite their potential for generating inaccurate information, or “hallucinations”. Their use is also expanding in the dental field. However, there are limited studies that provide objective evaluations of their performance. For the first time, we have compared the performance of students and four LLM-based chatbots in the field of oral and maxillofacial radiology.

Our study evaluated the performance of OpenAI’s ChatGPT and ChatGPT Plus, Google Bard, and Microsoft Bing Chat on regular examinations from oral and maxillofacial radiology courses at a dental college. This study was conducted before the release of GPT-4V (GPT-4 with vision), which has the capability to process images, so we excluded questions containing images such as radiographs, clinical photographs, and illustrations. Unlike previous studies,^5^^,^^8^^,^^9^^,^^11^^,^^12^^,^^14–17^ our research did not utilize an official test corpus such as the USMLE, so determining the passing rate was not possible. Instead, we used exam questions administered at a dental college to compare students' accuracy rates and the performance of the four chatbots.

The performance of each chatbot was lower than the students’ results (81.2%), not supporting the research hypothesis: ChatGPT Plus, 65.4%; Bing Chat, 63.5%; and ChatGPT and Bard, 50.0%. These results also fell short of the highest scores achieved on exam questions in the medical field (82.6% in neurosurgery,^10^ 87.11% in radiology,^11^ and 70.8% in respiratory medicine^13^) and dental field (76.9% in general dentistry,^14^ 73.6% in periodontology,^17^ and 90.63% in oral and maxillofacial surgery^20^). Previous studies^5^^,^^8–21^ have shown that the performance of LLM-based chatbots in the medical and dental fields varies significantly depending on the examinations and specific domains, but ChatGPT Plus generally exhibited the highest performance, consistent with our research findings.^10^^,^^12^^,^^14^^,^^15^^,^^17^^,^^18^

Regarding the questions’ educational content, students had the highest accuracy rate (83.5%) for imaging and equipment, and a similar accuracy rate of approximately 79% for basic knowledge and image interpretation. In basic knowledge, ChatGPT Plus was the only chatbot that exceeded the students' accuracy rate (93.8%). In image interpretation, all chatbots showed an accuracy of less than 35%. This indicates that chatbots are heavily trained on general content, but special domains would require training with specific dental data.

With regard to the question type, students had similar accuracy rates for MCQs (80.5%) and SAQs (82.9%), but all four chatbots showed higher accuracy rates for SAQs. In particular, ChatGPT Plus scored 85.7% for SAQs. For MCQs, all chatbots showed an accuracy of less than 60%. The SAQs used in this study necessitated clear and straightforward answers that focused on key concepts, whereas the MCQs required an accurate assessment of all presented options, necessitating extensive and detailed knowledge of oral and maxillofacial radiology. The simpler nature of SAQs likely contributed to their lower difficulty level compared to MCQs, leading to a higher accuracy rate.

In our study, we only calculated the correct answer rate without considering the validity of the answers. Often, when chatbots provided the correct answer, it was either unsupported by a rationale or resulted from AI hallucination, which produced a seemingly plausible answer. For instance, in an MCQ about performing periapical radiography at a different vertical angle after root canal treatment, the chatbot suggested that radiography at various angles might be necessary. However, the more accurate explanation involves adjusting the horizontal angle, not the vertical angle.

This study has several limitations, including the small number of test questions and the exclusion of questions that incorporate images. Future research should involve a larger set of questions covering various dental components. Additionally, as multimodal AI technology that incorporates images continues to advance, it will also be necessary to analyse questions that include images.

## Conclusions

Four commonly used LLM-based chatbots exhibited subpar performance in oral and maxillofacial radiology, particularly in areas that required specialized knowledge. They showed low accuracy and frequent AI hallucinations, even when their responses were correct. This underscores the importance of training models with high-quality data for their future use in oral and maxillofacial radiology.

## Supplementary Material

twae021_Supplementary_Data
