# Supplementary material for: How well do large language model-based chatbots perform in oral and maxillofacial radiology?
Source: Dentomaxillofac Radiol. 2024 Jun 7;53(6):390–5. doi: 10.1093/dmfr/twae021 (PMC11358622; doi:10.1093/dmfr/twae021)
Supplement: twae021_Supplementary_Data [file twae021_supplementary_data.docx]

**Appendix 1.** Original Korean and English translations of 52 oral and maxillofacial radiology exam questions used in this study.

| **Input prompt** | **Answer** |
| --- | --- |
| - **Basic Knowledge (n=16)** |  |
| - **Multiple-choice questions (n=14)** |  |
| **1. 사람에 미치는 방사선의 위험도를 평가하기 위하여 사용하는 방사선측정단위로, 조직에 따라 방사선감수성이 다른 것을 고려한 것으로 가장 옳은 것을 한 가지만 고르시오.** | 2 |
| (Choose the most accurate statement regarding the unit of radiation measurement used to evaluate the risk of radiation to humans considering that tissues differ in terms of radiation sensitivity) |  |
| ① 방사능 |  |
| (Radioactivity) |  |
| ② 유효선량 |  |
| (Effective dose) |  |
| ③ 조사선량 |  |
| (Exposure dose) |  |
| ④ 흡수선량 |  |
| (Absorbed dose) |  |
| ⑤ 등가선량 |  |
| (Equivalent dose) |  |
| **2. 방사선 생물학에 대한 설명으로 가장 옳은 것을 한 가지만 고르시오.** | 2 |
| (Choose the most accurate statement about radiobiology.) |  |
| ① 수정체는 고감수성이고, 림프조직은 저감수성이다. |  |
| (The lens of the eye is highly sensitive to radiation, while lymphatic tissue is less sensitive.) |  |
| ② 사망에 가장 큰 영향을 준 조직을 결정조직이라고 한다. |  |
| (The tissue with the most significant impact on mortality is known as critical tissue.) |  |
| ③ G1기에 방사선 조사가 되면 염색분체형 이상이 발생한다. |  |
| (Radiation exposure during the G1 phase can cause chromatid-type aberrations.) |  |
| ④ 세포질이 핵보다 방사선 감수성이 더 높으며, 형태적, 기능적으로 미분화 된 세포에서 방사선 감수성이 더 높다. |  |
| (The cytoplasm is more sensitive to radiation than the nucleus, and radiation sensitivity is higher in morphologically and functionally undifferentiated cells.) |  |
| ⑤ 광자가 생체를 구성하는 물 분자의 전리과정의 산물로 생성된 자유라디칼에 의하여 생체분자의 변화를 발생시키는 것은 직접효과이다. |  |
| (Photons cause changes in biomolecules through direct effects produced by the ionization of water molecules in the body, resulting in the generation of free radicals.) |  |
| **3. 방사선 방어에 대한 설명으로 가장 옳은 것을 한 가지만 고르시오.** | 2 |
| (Choose the most accurate statement about radiation protection.) |  |
| ① 방사선 장비 선택은 원형 조사통이 직사각형 조사통보다 좋다. |  |
| (When selecting radiation equipment, a circular collimator is preferable to a rectangular collimator.) |  |
| ② 확정적 영향의 발생은 방지하고, 확률적 영향의 발생을 최소로 하는 것이 목표이다. |  |
| (The goal is to prevent deterministic effects and minimize stochastic effects.) |  |
| ③ 일반 대중의 유효선량 한도는 50mSv/년이고 특수한 상황에서는 어느 한 해에 높은 선량이 허용된다. |  |
| (The effective dose limit for the general public is 50 mSv per year, and higher doses are allowed in special circumstances in a specific year.) |  |
| ④ 방어최적화는 개인 또는 사회가 방사선 노출에 의하여 얻을 수 있는 진단 정보가 방사선에 의하여 야기되는 상해보다 충분히 커야 한다는 것이다. |  |
| (Radiation protection optimization involves ensuring that the diagnostic information obtained from radiation exposure outweighs the potential harm caused by radiation-induced injury.) |  |
| ⑤ 촬영자는 환자로부터 최소한 1.8m 이상 떨어져 있어야 하고, x선속의 중심선에 대해 135~180° 사이가 좋다. |  |
| (The operator must maintain a minimum distance of 1.8 meters from the patient, and an angle of 135 to 180 degrees relative to the central axis of the X-ray beam is preferable.) |  |
| **4. 다음 중 가장 옳은 것을 한 가지만 고르시오.** | 5 |
| (Choose the most accurate statement from the following options.) |  |
| ① 전리방사선은 알파선, 베타선, 중성자선, 적외선, X선이 포함된다. |  |
| (Ionizing radiation includes alpha rays, beta rays, neutron rays, infrared rays, and X-rays.) |  |
| ② 양성자와 중성자를 합한 개수를 원자번호라 한다. |  |
| (The sum of protons and neutrons is called the atomic number.) |  |
| ③ 알파선은 보통 종이 1장으로도 차단시킬 수 있으므로 인체 위험성이 적다. |  |
| (Alpha rays usually can be blocked by a single sheet of paper, making them less hazardous to humans.) |  |
| ④ 음전하는 핵 주위를 빛의 속도로 돌고 있는데 핵에서 먼 위치일수록 결합에너지가 크다. |  |
| (Electrons orbit the nucleus at the speed of light, and the farther they are from the nucleus, the greater their energy.) |  |
| ⑤ 동위원소는 화학적 성질은 같으나 물리적 성질은 다르다. |  |
| (Isotopes have the same chemical properties but differ in their physical properties.) |  |
| **5. 전자기방사선에 대한 설명으로 가장 옳은 것을 한 가지만 고르시오.** | 1 |
| (Choose the most accurate statement about electromagnetic radiation.) |  |
| ① 감마선, X선, 자외선, 가시광선, 라디오파가 속한다. |  |
| (Gamma rays, X-rays, ultraviolet light, visible light, and radio waves are all types of electromagnetic radiation.) |  |
| ② 사인(sine)곡선을 그리며 전파하며 전기장과 자기장에 평행하게 진행한다. |  |
| (Electromagnetic radiation propagates in a sine wave pattern and travels parallel to the electric and magnetic fields.) |  |
| ③ 입자설은 굴절, 반사, 회절, 간섭, 편광 등의 현상을 설명하는 데 유용하다. |  |
| (The particle theory is useful for explaining phenomena such as refraction, reflection, diffraction, interference, and polarization.) |  |
| ④ 광자는 빛의 속도로 움직이며 에너지가 없는 질량체이다. |  |
| (Photons travel at the speed of light and are massless particles with no energy.) |  |
| ⑤ 입자의 전하가 클수록, 속도가 빠를수록 더 많은 에너지 전달이 가능하다. |  |
| (The greater a particle’s charge and speed, the more energy it can transfer.) |  |
| **6. X선이 가시광선과 다른 특징으로 가장 옳은 것을 한 가지만 고르시오.** | 2 |
| (Choose the most accurate statement about a characteristic that differentiates X-rays from visible light.) |  |
| ① 직진한다. |  |
| (They travel in a straight line.) |  |
| ② 원자를 전리(이온화)시킬 수 있다. |  |
| (They can ionize atoms.) |  |
| ③ 전장이나 자장에 의해 굴절되지 않는다. |  |
| (They are not refracted by electric or magnetic fields.) |  |
| ④ X선 필름에 대한 감광작용이 있다. |  |
| (They have a photosensitizing effect on X-ray film.) |  |
| ⑤ 초당 약 30만km를 전파한다. |  |
| (They propagate at about 300,000 km per second.) |  |
| **7. X선 양극에서 발생하는 원리에 대한 설명으로 가장 옳은 것을 한 가지만 고르시오.** | 3 |
| (Choose the most accurate statement regarding the principles of operation at the X-ray anode.) |  |
| ① 단위면적당 발생하는 열을 줄이기 위해 초점 크기를 감소시킨다. |  |
| (To reduce heat generation per unit area, the focus size is decreased.) |  |
| ② 실초점(actual focal spot)을 실효초점(effective focal spot)보다 작게 되도록 하는 것이 선초점의 원리이다. |  |
| (The line focus principle involves making the actual focal spot smaller than the effective focal spot.) |  |
| ③ 양극의 열을 제거하기 위해 구리봉을 사용한다. |  |
| (Copper rods are used to dissipate heat from the anode.) |  |
| ④ 타깃은 원자번호가 작을수록 유리하다. |  |
| (A smaller atomic number is advantageous for the target.) |  |
| ⑤ 텅스텐 타깃은 증기압이 높아 진공상태를 유지시키기 용이하다. |  |
| (A tungsten target has high vapor pressure, making it easy to maintain a vacuum.) |  |
| **8. X선속에 대한 설명으로 가장 옳은 것을 한 가지만 고르시오.** | 4 |
| (Choose the most accurate statement about X-ray beams.) |  |
| ① 임상에서 관전압을 증가시키고 동일한 흑화도를 얻으려면 노출시간을 증가시켜야 한다. |  |
| (In clinical practice, increasing the tube voltage while maintaining the same level of density requires increasing the exposure time.) |  |
| ② 반가층이 클수록 X선속의 평균 에너지는 낮아진다. |  |
| (The higher the half-value layer, the lower the average energy of the X-ray beam.) |  |
| ③ 관전류를 높이면 X선 광자의 수, 광자의 평균 에너지, 최대 에너지 모두 증가한다. |  |
| (Increasing the tube current increases the number of X-ray photons, their average energy, and their maximum energy.) |  |
| ④ 다색 방사선이 여과기를 통과하면 X선의 평균 에너지가 증가한다. |  |
| (When polyenergetic radiation passes through a filter, the average energy of the X-ray beam increases.) |  |
| ⑤ X선속의 강도는 초점으로부터의 거리제곱에 비례한다. |  |
| (The intensity of the X-ray beam is proportional to the square of the distance from the focal spot.) |  |
| **9. X선과 물질의 상호작용과 그에 대한 설명으로 가장 옳은 것을 한 가지만 고르시오.** | 1 |
| (Choose the most accurate statement about the interaction between X-rays and matter.) |  |
| ① 광전효과는 원자번호가 높을수록 발생확률이 높아지며 흑화도의 차이로 나타난다. |  |
| (The probability of the photoelectric effect increases with higher atomic numbers and is reflected in differences in density.) |  |
| ② 고전산란은 입사한 X선 광자의 진행 방향이 바뀌며 전자를 전리시킨다. |  |
| (Classical scattering occurs when the direction of an incident X-ray photon changes, causing the ionization of electrons.) |  |
| ③ 콤프턴 산란은 산란선을 발생시키지 않아 영상의 질을 향상시켜 술자에게 유리하다. |  |
| (Compton scattering does not produce scatter radiation, which enhances image quality, making it advantageous for practitioners.) |  |
| ④ 광전효과는 입사광자의 에너지 일부가 조직 밖으로 빠져나가므로 환자에게 유리하다. |  |
| (The photoelectric effect is favorable for patients because a portion of the incident photon’s energy escapes from the tissue.) |  |
| ⑤ 고전산란은 전자밀도에 직접적으로 비례하여 연조직에서 더 많이 투과된다. |  |
| (Classical scattering is directly proportional to electron density and is more transmissive in soft tissues.) |  |
| **10. 방사선영상의 흑화도에 영향을 미치는 요인에 대한 설명으로 가장 옳은 것을 한 가지만 고르시오.** | 2 |
| (Choose the most accurate statement about factors influencing radiographic density.) |  |
| ① X선속 시준(collimation)은 흑화도와 밀접한 관계가 있다. |  |
| (X-ray beam collimation is closely related to density.) |  |
| ② 피사체 두께에 따른 감쇠를 보상하기 위해 X선 노출시간을 조절해야 한다. |  |
| (To compensate for attenuation due to object thickness, the X-ray exposure time must be adjusted.) |  |
| ③ 초점-필름 거리가 증가될수록 흑화도가 증가된다. |  |
| (As the focal spot-to-film distance increases, density also increases.) |  |
| ④ 노출시간의 증가는 흑화도보다는 대조도에 영향을 미친다. |  |
| (An increase in exposure time impacts contrast more than density.) |  |
| ⑤ 부가여과량을 늘리면 흑화도가 증가된다. |  |
| (Increasing additional filtration leads to an increase in density.) |  |
| **11. 방사선 영상의 특성에 대한 설명으로 가장 옳은 것을 한 가지만 고르시오.** | 1 |
| (Choose the most accurate statement about the characteristics of radiographic images.) |  |
| ① 흑화도와 노출량의 관계를 나타내는 특성곡선의 기울기가 클수록 필름 대조도가 높다. |  |
| (The steeper the slope of the characteristic curve representing the relationship between density and exposure, the higher the film’s contrast.) |  |
| ② 대조도는 구별 가능한 흑화도로 기록될 수 있는 노출의 범위를 뜻한다. |  |
| (Contrast refers to the range of exposure that can be recorded as distinguishable densities.) |  |
| ③ 높은 관전압으로 촬영하면 동일한 흑화도를 얻을 때 대조도가 높아진다. |  |
| (Higher tube voltage leads to greater contrast at the same density.) |  |
| ④ 선예도는 초점-피사체 거리 감소, 피사체-상수용기 거리를 증가시키면 향상된다. |  |
| (Sharpness improves when the focal spot-to-object distance decreases and the object-to-image receptor distance increases.) |  |
| ⑤ 산란선은 높은 관전압 사용, 부과 여과 사용 등으로 감소시킬 수 있다. |  |
| (Scatter radiation can be reduced by using high tube voltage and additional filtration.) |  |
| **12. 디지털 영상에 대한 설명으로 가장 옳은 것을 한 가지만 고르시오.** | 4 |
| (Choose the most accurate statement about digital imaging.) |  |
| ① 아날로그 영상은 작은 크기로 분할된 회색조 정보가 단계별로 표현된다. |  |
| (An analog image is divided into smaller segments of grayscale information presented in a stepwise manner.) |  |
| ② X선이 피사체를 통과하고 검출기의 광섬유판이 가시광선으로 변환시킨다. |  |
| (After X-rays pass through an object, the optical fiber plate of the detector converts them into visible light.) |  |
| ③ CCD 방식은 독립적으로 각 화소가 트랜지스터와 직접 연결된 방식으로 저렴하다. |  |
| (The CCD method uses an independent system where each pixel is directly connected to a transistor, making it cost-effective.) |  |
| ④ 비트 수가 높으면 미세한 회색조의 차이를 감지하는데 유리하다. |  |
| (A higher bit depth is beneficial for detecting subtle variations in grayscale.) |  |
| ⑤ SNR (signal to noise ratio)이 높으면 노이즈가 상대적으로 높음을 의미한다. |  |
| (A high signal-to-noise ratio (SNR) indicates that noise is relatively high.) |  |
| **13. 디지털 영상에서 화소의 크기와 공간 해상도가 가장 옳게 연결된 것을 한 가지만 고르시오.** | 4 |
| (Choose the most accurate statement about the correct association between pixel size and spatial resolution in digital imaging.) |  |
| ① 10 µm: 25 lp/mm |  |
| ② 20 µm: 50 lp/mm |  |
| ③ 25 µm: 10 lp/mm |  |
| ④ 100 µm: 5 lp/mm |  |
| ⑤ 500 µm: 2 lp/mm |  |
| **14. 포렌식 분석법에서 치과의료영상의 활용에 대한 설명으로 가장 옳지 않은 것을 한 가지만 고르시오.** | 1 |
| (Choose the most inaccurate statement about the use of dental radiographic imaging in forensic analysis.) |  |
| ① 생전 영상기록(antemortem record)이 없다면 활용도가 적다. |  |
| (If there are no antemortem records, its utility is limited.) |  |
| ② 사후 시편을 손상시키지 않고 정보를 얻을 수 있는 비침습적 방법이다. |  |
| (It is a non-invasive method for obtaining information without damaging postmortem samples.) |  |
| ③ 연령감정 방법으로 제3대구치의 발달평가를 기반으로 한 Demirjian’s criteria가 확립되었다. |  |
| (Demirjian’s criteria, based on the development of the third molar, has been established as an age estimation method.) |  |
| ④ 파노라마에서 연령이 증가할수록 치아의 2차 상아질이 증가하는 점을 활용하기도 한다. |  |
| (Panoramic radiographs can utilize the increase in secondary dentin with age to help in age estimation.) |  |
| ⑤ 대규모 재난에서 개인 식별 방법으로 활용되기도 한다. |  |
| (It is also used as a method for personal identification in large-scale disasters.) |  |
| - **Basic Knowledge (n=16)** |  |
| - **Short-answer questions (n=2)** |  |
| **15. 다음 제시글의 빈 칸에 알맞은 용어를 쓰시오.** | 진단참고준위  (Diagnostic reference levels (DRLs)) |
| (Fill in the blank with the correct term.) |  |
| ( )은 1996년 ICRP에서 국가나 지역의 의료기관에서 시행하고 있는 방사선검사 및 핵의학 검사의 방사선량을 조사하여 백분위로 표시한 후, 기준이 되는 방사선량을 제시한 것으로 전국 의료기관에서 진단영상검사의 촬영조건과 피폭선량을 조사하여 환자 피폭선량 분포의 3사분위 값으로 설정하는 것이다. |  |
| ( ) was introduced by the International Commission on Radiological Protection (ICRP) in 1996, where the radiation doses from radiological and nuclear medicine examinations conducted at national or regional medical institutions are surveyed and represented in percentiles, followed by presenting a reference radiation dose. This involves investigating the imaging conditions and radiation doses for diagnostic imaging at medical institutions nationwide, then establishing the reference level at 75^th^ percentile of the distribution of patient exposure doses.) |  |
| **16. 다음 제시글의 빈 칸에 알맞은 용어를 쓰시오.** | 반감기  (Half-life) |
| (Fill in the blank with the correct term.) |  |
| 방사선 물질의 수명은 ( )로 표현되는데, 이것은 처음에 존재한 방사선동위원소의 원자 수가 방사성붕괴에 의해 본래의 절반이 될 때까지 소요되는 시간을 의미한다. |  |
| (The lifespan of radioactive substances is expressed in ( ). This term refers to the time it takes for the number of atoms of a radioactive isotope to be reduced by one-half due to radioactive decay.) |  |
| - **Imaging and equipment (n=27)** |  |
| - **Multiple-choice questions (n=16)** |  |
| **17. 방사선 영상의 상의 질을 높이는 방법으로 가장 옳은 것을 한 가지만 고르시오.** | 3 |
| (Choose the most accurate statement regarding how to improve the quality of radiographic images.) |  |
| ① 초점을 크게 하여 선예도를 증가시킨다. |  |
| (Increase the focal spot size to enhance sharpness.) |  |
| ② 초점-피사체 거리를 감소시키고, 피사체-상수용기 거리를 증가시킨다. |  |
| (Decrease the focal spot-to-object distance and increase the object-to-image receptor distance.) |  |
| ③ 낮은 관전압, X선속의 시준과 격자를 통해 산란선을 감소시킨다. |  |
| (Reduce scatter radiation by using low tube voltage, X-ray beam collimation, and grids.) |  |
| ④ 동일한 흑화도에서 높은 관전압을 사용하여 대조도가 높은 영상을 얻는다. |  |
| (Use high tube voltage to obtain high-contrast images with the same density.) |  |
| **18. 구내방사선촬영술에 대한 설명으로 가장 옳은 것을 한 가지만 고르시오.** | 4 |
| (Choose the most accurate statement about intraoral radiography.) |  |
| ① 상악 치아를 촬영하는 경우 하악 치아 촬영 시보다 고개를 들고 촬영한다. |  |
| (When imaging maxillary teeth, the head is tilted upward more than when imaging mandibular teeth.) |  |
| ② 중심선의 수평각은 관구를 상하로 움직이며 조절한다. |  |
| (The horizontal angle of the central ray is adjusted by moving the tube head up and down.) |  |
| ③ 관구가 천장을 향하는 경우 중심선은 (+) 각도를 보인다. |  |
| (When the tube head is pointing toward the ceiling, the central ray shows a positive angle.) |  |
| ④ 방사선조사야는 환자의 피부에서 직경이 7cm를 초과하여서는 안 된다. |  |
| (The X-ray beam diameter at the patient’s skin should not exceed 7 cm.) |  |
| ⑤ 갑상선보호대가 있는 납방어복을 착용할 필요는 없다. |  |
| (There is no need to wear a lead apron with a thyroid shield.) |  |
| **19. 구내방사선영상의 화질과 관련된 설명으로 가장 옳은 것을 한 가지만 고르시오.** | 4 |
| (Choose the most accurate statement about the quality of intraoral radiographic images.) |  |
| ① 상의 선예도와 해상도는 초점-피사체의 거리가 가까울수록 높아진다. |  |
| (The sharpness and resolution of the image increase as the focal spot-to-object distance becomes shorter.) |  |
| ② 필름과 피사체 사이 거리가 가까울수록 상의 확대율이 낮다. |  |
| (The closer the distance between the film and the object, the lower the magnification rate of the image.) |  |
| ③ 초점과 피사체가 평행하게 위치되어야 상의 크기 왜곡이 없다. |  |
| (The image size has no distortion when the focal spot and object are positioned parallel.) |  |
| ④ 실효초점이 작을수록 반음영이 적게 형성되며 화질이 증가한다. |  |
| (The smaller the effective focal spot, the smaller the penumbra, leading to better image quality.) |  |
| **20. 구내방사선영상에 대한 설명으로 가장 옳은 것을 한 가지만 고르시오.** | 4 |
| (Choose the most accurate statement about intraoral radiography.) |  |
| ① 혼합치열기 초기에는 치근단방사선검사는 필요하지 않다. |  |
| (During the early mixed dentition stage, periapical radiographs are not necessary.) |  |
| ② 10~15세 환자의 경우 성인과 동일한 수준의 노출량을 사용한다. |  |
| (Patients aged 10 to 15 require the same exposure level as adults.) |  |
| ③ 근관치료 후 검사 시 수직각을 변화한 치근단방사선검사를 시행한다. |  |
| (When examining after root canal treatment, periapical radiographs with different vertical angles are used.) |  |
| ④ 개구가 어려운 환자의 경우 교합방사선촬영을 시행할 수 있다. |  |
| (For patients with difficulty opening their mouths, occlusal radiography may be used.) |  |
| **21. 구외방사선촬영술에 대한 설명으로 가장 옳은 것을 한 가지만 고르시오.** | 2 |
| (Choose the most accurate statement about extraoral radiography.) |  |
| ① 후전방두부방사선촬영술에서 X-ray 중심선은 환자의 외이공을 통과한다. |  |
| (In postero-anterior cephalometric radiography, the central X-ray beam passes through the patient’s external auditory meatus.) |  |
| ② 후전방두부방사선촬영술을 통해 안모의 측방성장을 평가할 수 있다. |  |
| (Postero-anterior cephalometric radiography can be used to evaluate the lateral growth of the facial structure.) |  |
| ③ 워터스방사선촬영술 시행 시 개구 상태를 유지한다. |  |
| (In Waters radiographic technique, the patient’s mouth should be open during the exposure.) |  |
| ④ 하악과두가 골절되어 변위된 경우 역타운방사선영상을 통한 관찰은 어렵다. |  |
| (Observing displaced mandibular condyle fracture with reverse Towne’s radiographs is difficult.) |  |
| ⑤ 이하두정방사선영상은 측두골의 추체부 관찰을 목적으로 하는 촬영술이다. |  |
| (A submentovertex radiograph is intended to visualize the petrous part of the temporal bone.) |  |
| **22. 구내방사선영상과 비교한 파노라마방사선영상의 장점으로 가장 옳지 않은 것을 한 가지만 고르시오.** | 4 |
| (Choose the most inaccurate statement about the advantages of panoramic radiography compared to intraoral radiography.) |  |
| ① 상하악골 전반에 걸쳐 1장의 영상으로 관찰할 수 있다. |  |
| (It allows observation of both the maxilla and mandible in a single image.) |  |
| ② 촬영이 간편하다. |  |
| (It is easy to take.) |  |
| ③ 촬영 시간이 짧다. |  |
| (The exposure time is short.) |  |
| ④ 안면부 전반에 걸쳐 X선 노출이 많다. |  |
| (It results in high X-ray exposure across the entire facial area.) |  |
| ⑤ 영상의 해상도가 비교적 높다. |  |
| (The resolution of the image is relatively high.) |  |
| **23. 파노라마방사선촬영술에 대한 설명으로 가장 옳은 것을 한 가지만 고르시오.** | 4 |
| (Choose the most accurate statement about panoramic radiography.) |  |
| ① 방사선원은 가로로 길이가 긴 슬릿 형태이다. |  |
| (The X-ray source is in the form of a horizontally elongated slit.) |  |
| ② 회전중심과 방사선원 사이에 위치하는 구조물은 실상으로 형성된다. |  |
| (Structures located between the rotation center and the X-ray source appear as real images.) |  |
| ③ 영상에서 실상이 2번 허상이 1번 동시에 형성되는 경우를 촬영오류라고 한다. |  |
| (An imaging error occurs when two real images and one ghost image are formed simultaneously.) |  |
| ④ 허상은 구조물과 동일한 형태로 반대측 상방에 형성된다. |  |
| (Ghost images appear on the opposite side and above the original structure.) |  |
| ⑤ 귀걸이를 착용하고 촬영한 경우 영상에서 허상으로 나타나는 경우는 드물다. |  |
| (It is rare for ghost images to occur when earrings are worn during the imaging process.) |  |
| **24. 단층촬영술에 대한 설명으로 가장 옳지 않은 것을 한 가지만 고르시오.** | 1 |
| (Choose the most inaccurate statement about tomographic imaging.) |  |
| ① 단층촬영각이 작을수록 초점층의 두께는 얇아진다. |  |
| (The smaller the tomographic angle, the thinner the focal plane.) |  |
| ② X선관두, X선필름 및 연결대로 구성되며 X선관두와 필름이 고정축을 중심으로 동시에 반대 방향으로 이동한다. |  |
| (A tomographic imaging device consists of an X-ray tube head, X-ray film, and a linkage arm, with the X-ray tube head and film moving simultaneously in opposite directions around a fixed axis.) |  |
| ③ 초점층 외부에 위치한 구조물은 흐린 상을 형성하고 초점층의 상을 더욱 선명하게 보여주는 방사선촬영술이다. |  |
| (Structures located outside the focal plane form blurred images, enhancing the sharpness of the images within the focal plane.) |  |
| ④ 측두하악관절 및 임플란트를 위한 악골의 절단면 관찰 목적으로 사용되었으나 콘빔 CT, CT, MRI 등의 사용으로 사용이 감소하였다. |  |
| (Tomographic imaging was previously used to observe cross-sections of temporomandibular joint and dental implants, but its use has decreased due to the adoption of cone-beam CT, CT, and MRI.) |  |
| ⑤ X선관두는 나선, 타원 및 클로버 잎 등의 모양으로 이동하며 이동이 복잡할수록 선명한 상을 얻을 수 있다. |  |
| (The X-ray tube head moves in shapes such as spirals, ellipses, and clovers, and the more complex the movement, the clearer the image obtained.) |  |
| **25. 자기공명영상에 대한 설명으로 가장 옳은 것을 한 가지만 고르시오.** | 4 |
| (Choose the most accurate statement about magnetic resonance imaging.) |  |
| ① 전리방사선을 사용하며 촬영 시간이 길고 폐쇄공포증 환자에서 시행이 어렵다. |  |
| (It uses ionizing radiation, takes a long time, and is challenging for patients with claustrophobia.) |  |
| ② 체내에 분포하는 원자 중 그 번호가 홀수인 수소, 탄소, 불소 등이 자기핵에 해당하고 대부분의 영상진단은 그 중 가장 높은 자성을 띠는 탄소 양성자를 사용한다. |  |
| (Among atoms in the body with odd atomic numbers, such as hydrogen, carbon, and fluorine, the majority of imaging diagnostics use carbon protons, which have the highest magnetism.) |  |
| ③ RF 펄스는 공명 현상을 일으켜 횡축자기화가 감소되고 종축자기화가 발생한다. |  |
| (RF (radiofrequency) pulses cause resonance, resulting in decreased transverse magnetization and increased longitudinal magnetization.) |  |
| ④ T1강조영상은 지방이 잘 보이고 T2강조영상은 물이 밝게 나타난다. |  |
| (T1-weighted images show fat clearly, while T2-weighted images depict water more brightly.) |  |
| ⑤ T1강조영상은 염증, 종양 등 병적 소견 판독에 유용하고 T2강조영상은 정상해부학적 구조 판별에 유용하다. |  |
| (T1-weighted images are useful for interpreting pathological findings like inflammation and tumors, while T2-weighted images are beneficial for distinguishing normal anatomical structures.) |  |
| **26. 특수진단영상에 대한 설명으로 가장 옳은 것을 한 가지만 고르시오.** | 5 |
| (Choose the most accurate statement about special diagnostic imaging.) |  |
| ① 조영촬영술에 사용되는 조영제는 주로 지용성 조영제이다. |  |
| (The contrast agent used in contrast radiography is primarily a fat-soluble contrast agent.) |  |
| ② 타액선에 급성 염증이 있을 때에는 타액선 스캔보다는 타액선 조영술이 유용하다. |  |
| (Sialography is more useful than a salivary gland scan when there is acute inflammation in the salivary glands.) |  |
| ③ 초음파검사는 골조직을 잘 통과하여 심부를 검사할 수 있다. |  |
| (Ultrasound can penetrate bone structures, allowing deep examinations.) |  |
| ④ 타액선 스캔은 주타액선의 기능 정보를 제공하고 쇼그렌증후군은 열점으로 나타난다. |  |
| (Salivary gland scans provide functional information about the major salivary glands, and Sjogren’s syndrome appears as hot spots.) |  |
| ⑤ 핵의학검사 시 사용되는 방사선동위원소로 Technetium이 타액선, 갑상선, 골스캔 등에 가장 많이 사용된다. |  |
| (The most commonly used radioisotope in nuclear medicine is technetium, which is frequently utilized for salivary gland, thyroid, and bone scans.) |  |
| **27. 방사선 촬영 장비 및 관련 기기의 관리에 대한 설명으로 가장 옳은 것을 한 가지만 고르시오.** | 4 |
| (Choose the most accurate statement about managing radiographic equipment and related devices.) |  |
| ① 관전류는 금속 회전탑으로 측정 가능하다. |  |
| (Tube current can be measured using a metal rotating tower.) |  |
| ② 차폐 시설은 0.5~1.0mm 두께의 납 또는 이에 해당하는 석고, 콘크리트, 타일, 벽돌 또는 강철 등을 사용한다. |  |
| (Shielding facilities use 0.5 to 1.0-mm-thick lead or equivalent materials like gypsum, concrete, tile, brick, or steel.) |  |
| ③ 반파 자기 정류 방사선 촬영장비는 일정한 전압이 유지되어 짧은 촬영 시간에 영상을 얻을 수 있으나 고가라는 단점이 있다. |  |
| (Half-wave self-rectifying radiographic equipment maintains a constant voltage, allowing short exposure times, but is expensive.) |  |
| ④ 진단용 방사선발생장치의 안전관리에 관한 규칙에 의거하여 사용 3일전까지 검사기관의 검사를 받고 3년마다 정기 검사를 시행한다. |  |
| (According to safety management rules for diagnostic radiographic devices, the equipment must be inspected by a testing agency 3 days before use and every 3 years for regular inspections.) |  |
| ⑤ X선속의 강도를 ½로 감소시키는 데 필요한 물질의 두께를 반가층이라 하고 통상적으로 납 두께로 표시한다. |  |
| (The thickness of the material needed to reduce the intensity of the X-ray beam by half is called the half-value layer and is commonly represented in terms of lead thickness.) |  |
| **28. 치과용 포터블 방사선촬영장치 사용 시 권고사항에 대한 설명으로 가장 옳지 않은 것을 한 가지만 고르시오.** | 2 |
| (Choose the most inaccurate statement about the recommended guidelines for using portable dental X-ray machines.) |  |
| ① 후방 산란성 차폐제가 부착된 촬영장치를 사용하는 것이 좋다. |  |
| (It is recommended to use an X-ray machine equipped with a rear scatter shield.) |  |
| ② 가급적 초점-피부 간 거리가 짧은 X선 촬영장치를 사용한다. |  |
| (Portable X-ray machines with a short focal spot-to-skin distance are preferred.) |  |
| ③ 필요한 상황이 아니면 기존의 고정형 구내 방사선 촬영기를 사용한다. |  |
| (Unless necessary, existing fixed intraoral X-ray units should be used.) |  |
| ④ 환자에 대한 보호보다 술자의 안전에 특히 주의한다. |  |
| (More attention should be paid to operator safety than to patient protection.) |  |
| ⑤ 촬영자는 개인 피폭선량계, 진료용 X선 방어앞치마, 갑상선보호대, 방어용 납장갑 등을 반드시 착용하고 촬영한다. |  |
| (The operator must wear a personal dosimeter, lead apron, thyroid shield, and protective lead gloves during imaging.) |  |
| **29. 감염방지에 대한 설명으로 가장 옳은 것을 한 가지만 고르시오.** | 3 |
| (Choose the most accurate statement about infection prevention.) |  |
| ① 차단용품이 표면청결과 표면소독을 대신할 수 있다. |  |
| (Barrier products can be substituted for surface cleaning and disinfection.) |  |
| ② 구내용 디지털 센서는 가압멸균 또는 2% glutaraldehyde용액을 사용한다. |  |
| (Intraoral digital sensors are sterilized using an autoclave or a 2% glutaraldehyde solution.) |  |
| ③ 모든 환자들이 감염원이 될 수 있다고 가정하고 감염방지를 해야 한다. |  |
| (All patients should be assumed to be potential infection sources, and infection prevention measures should be followed.) |  |
| ④ 촬영 중에는 항상 일회용 라텍스 글러브나 비닐 글러브를 착용하고 정확한 촬영을 위해 환자의 구강내 타액과 접촉되는 것이 좋다. |  |
| (During imaging, one must always wear disposable latex or vinyl gloves, and contact with oral saliva is encouraged for accurate imaging.) |  |
| ⑤ 표면소독은 소독액이 가급적 장시간 표면에 묻어 있도록 해야 하고 오염도가 높은 부분에서 적은 부분으로 시행한다. |  |
| (Surface disinfection should be done to ensure the disinfectant remains on the surface for as long as possible, starting from highly contaminated areas and moving to less contaminated areas.) |  |
| **30. 콘빔시티 촬영장비의 기본 원리에 대한 설명으로 가장 옳은 것을 한 가지만 고르시오.** | 2 |
| (Choose the most accurate statement about the basic principles of cone-beam CT imaging equipment.) |  |
| ① 부채꼴의 X선속과 선원형 검출기를 사용한다. |  |
| (It uses a fan-shaped X-ray beam and a linear detector array.) |  |
| ② CT와 비교해서 연조직 대조도가 낮다. |  |
| (It has lower soft tissue contrast than conventional CT.) |  |
| ③ 파노라마 촬영장비보다 낮은 선량의 방사선을 활용한다. |  |
| (It uses lower radiation doses than panoramic imaging equipment.) |  |
| ④ 피폭량이 CT보다 적어 인공음영이 상대적으로 적게 발생한다. |  |
| (The radiation exposure is lower than with conventional CT, resulting in relatively fewer artifacts.) |  |
| ⑤ CT와 비교해서 촬영 시간이 길다. |  |
| (It takes longer to scan than conventional CT.) |  |
| **31. 콘빔시티의 영상 획득에 관한 설명으로 가장 옳은 것을 한 가지만 고르시오.** | 4 |
| (Choose the most accurate statement about cone-beam CT image acquisition.) |  |
| ① 검출기의 FOV (영상범위, field of view)는 모든 영상장비에서 동일하다. |  |
| (The field of view (FOV) of the detector is the same across all imaging equipment.) |  |
| ② 콘빔시티는 편판형 검출기 사용으로 인해 산란선이 CT보다 적은 편이다. |  |
| (Cone-beam CT has fewer scattered rays than conventional CT due to the use of flat-panel detectors.) |  |
| ③ 스캔 시 투사영상의 수가 많을수록 최종 영상의 노이즈가 증가한다. |  |
| (Increasing the number of projection images during scanning increases the noise in the final image.) |  |
| ④ 획득한 체적 데이터를 진단 목적에 따라 여러 단면으로 재구성한다. |  |
| (Acquired volumetric data can be reconstructed into various cross-sections depending on the diagnostic purpose.) |  |
| ⑤ 콘빔시티의 체적소는 직육면체이다. |  |
| (The voxel shape in cone-beam CT is cuboid.) |  |
| **32. 콘빔시티 영상을 생성하고 활용하는데 반드시 필요한 구성요소가 아닌 것으로 가장 옳은 것을 한 가지만 고르시오.** | 3 |
| (Choose the most accurate statement about a component that is not necessarily required to generate and utilize cone-beam CT images.) |  |
| ① X선 검출기 |  |
| (X-ray detector) |  |
| ② 영상 재구성 소프트웨어 |  |
| (Image reconstruction software) |  |
| ③ 그리드 |  |
| (Grid) |  |
| ④ 모니터 |  |
| (Monitor) |  |
| ⑤ 컴퓨터 |  |
| (Computer) |  |
| - **Imaging and equipment (n=27)** |  |

| - **Short-answer questions (n=11)** |  |
| --- | --- |

| **33. 제시글의 빈 칸에 알맞은 용어를 쓰시오.** | 작아야  (small) |
| --- | --- |
| (Fill in the blank with the correct term.) |  |
| 상투영의 5원칙에 따르면 방사선원은 가능한 한 ( ) 한다. |  |
| (According to the five principles of projection, the source of radiation should be as ( ) as possible.) |  |
| **34. 제시글의 빈 칸에 알맞은 용어를 쓰시오.** | 멀어야  (far) |
| (Fill in the blank with the correct term.) |  |
| 상투영의 5원칙에 따르면 방사선원과 피사체 간의 거리는 가능한 한 ( ) 한다. |  |
| (According to the five principles of projection, the distance between the radiation source and the object should be as ( ) as possible.) |  |
| **35. 제시글의 빈 칸에 알맞은 용어를 쓰시오.** | 짧아야  (short) |
| (Fill in the blank with the correct term.) |  |
| 상투영의 5원칙에 따르면 피사체와 필름 간의 거리는 가능한 한 ( ) 한다. |  |
| (According to the five principles of projection, the distance between the object and the film should be as ( ) as possible.) |  |
| **36. 제시글의 빈 칸에 알맞은 용어를 쓰시오.** | 평행  (parallel) |
| (Fill in the blank with the correct term.) |  |
| 상투영의 5원칙에 따르면 피사체와 필름은 가능한 한 ( )이 되어야 한다. |  |
| (According to the five principles of projection, the object and the film should be as ( ) as possible.) |  |
| **37. 제시글의 빈 칸에 알맞은 용어를 쓰시오.** | 수직  (vertical) |
| (Fill in the blank with the correct term.) |  |
| 상투영의 5원칙에 따르면 중심선은 피사체와 필름에 대해 가능한 한 각각 ( )으로 조사되어야 한다. |  |
| (According to the five principles of projection, the central ray should be projected as ( ) as possible with respect to the object and the film.) |  |
| **38-39. 제시글의 빈 칸에 알맞은 용어를 쓰시오.** | 체적소, 화소  (voxel, pixel) |
| (Fill in the blank with the correct term.) |  |
| CT 영상은 ( )라고 불리는 작은 육면체 구조들이 모여 구성된 메트릭스로 저장되어 관찰 가능한 영상으로 표현되고 이것이 CT 영상에 표현되는 2차원 사각형을 ( )라 한다. |  |
| (CT images are stored and represented as observable images in a matrix composed of small cubic structures called ( ), and the 2D rectangular elements depicted in CT images are called ( ).) |  |
| **40. 제시글의 빈 칸에 알맞은 용어를 쓰시오.** | 가돌리늄  (gadolinium) |
| (Fill in the blank with the correct term.) |  |
| MR 영상에서 사용되는 조영제의 성분은 ( )이다. |  |
| (The main ingredient of the contrast medium used in MR imaging is ( ).) |  |
| **41. 제시글의 빈 칸에 알맞은 용어를 쓰시오.** | 교차감염  (cross-infections) |
| (Fill in the blank with the correct term.) |  |
| 감염방지의 기본 목표는 환자와 환자, 그리고 환자와 술자 사이의 ( )을 방지하는 것이다. |  |
| (The primary goal of infection prevention is to prevent ( ) between patients, and between patients and practitioners.) |  |
| **42. 제시글의 빈 칸에 알맞은 용어를 쓰시오.** | 참고  (reference) |
| (Fill in the blank with the correct term.) |  |
| 좋은 화질의 ( ) 방사선영상을 비치해 두고 매일 촬영한 방사선 영상과 비교하여 흑화도, 대조도, 선명도 등 방사선영상의 질 변화를 평가하는 것이 좋다. |  |
| (It is recommended to obtain and keep a ( ) radiograph with high image quality and compare it daily with radiographs taken to evaluate changes in image quality, such as density, contrast, and sharpness.) |  |
| **43. 제시글의 빈 칸에 알맞은 용어를 쓰시오.** | ALARA  (as low as reasonably achievable) |
| (Fill in the blank with the correct term.) |  |
| 콘빔시티검사를 시행할 때에는 ( ) 원칙에 맞도록 환자의 신체 크기에 따라 노출 조건을 조절하여야 한다. |  |
| (When performing cone-beam CT, exposure conditions should be adjusted according to the patient’s body size to comply with the ( ) principle.) |  |
| - **Image interpretation (n=9)** |  |

| - **Multiple-choice questions (n=8)** |  |
| --- | --- |

| **44. 단순골낭(simple bone cyst)에 대한 설명으로 가장 옳은 것을 한 가지만 고르시오.** | 4 |
| --- | --- |
| (Choose the most accurate statement about simple bone cysts) |  |
| ① 주로 양측성으로 나타난다. |  |
| (They mainly appear bilaterally.) |  |
| ② 노년층에서 호발한다. |  |
| (They are prevalent in older people.) |  |
| ③ 병소 내 물결 모양의 격벽이 관찰되며 경계에서 내부로 직각으로 형성된 격벽이 특징이다. |  |
| (They display undulating septa within the lesion, with perpendicular septa extending inward from the border.) |  |
| ④ 이장상피가 없으며 병소 상방에서는 치근 사이로 확장된 조개껍데기 모양의 상연을 보인다. |  |
| (They lack a lining epithelium, and the superior portion of the lesion presents a shell-like edge expanding between the roots.) |  |
| ⑤ 병소에 의해 상악골 중앙 부위가 커져 보이는 경우가 있어 뼈사자얼굴이라고 불린다. |  |
| (Lesions might cause the central part of the maxilla to appear enlarged, sometimes termed “leontiasis ossea.”) |  |
| **45. 치아 외상에 대한 설명으로 가장 옳은 것을 한 가지만 고르시오.** | 4 |
| (Choose the most accurate statement about tooth trauma.) |  |
| ① 치아진탕은 치아의 비정상적인 동요와 변위를 보이며 치근단부의 치주인대강 확장 소견이 관찰된다. |  |
| (Tooth concussion displays abnormal mobility and displacement, with widening of the periodontal ligament space at the apex.) |  |
| ② 측방탈구된 치아는 외상을 받은 부위의 반대쪽에 치주인대강 확장 소견이 나타난다. |  |
| (Laterally luxated teeth often exhibit periodontal ligament space widening on the side opposite to the trauma.) |  |
| ③ 압입탈구된 치아의 치근이 함입된 위치에서의 치주인대강은 부분적으로 확장된 소견이 관찰된다. |  |
| (In intrusive luxated teeth, a partial widening of the periodontal ligament space at the location where the root is intruded is observed.) |  |
| ④ 치근 파절의 경우 파절면에 방사선의 중심선이 평행하게 조사되면 하나의 방사선투과성 선으로 나타난다. |  |
| (Root fractures appear as a single radiolucent line when the X-ray beam is parallel to the fracture line.) |  |
| ⑤ 치관 파절의 경우 방사선학적 소견이 정확한 상황을 묘사하므로 임상검사에서 획득한 정보는 고려하지 않아도 된다. |  |
| (Crown fractures can be accurately diagnosed with radiographic findings alone, making information from clinical examination unnecessary.) |  |
| **46. 구강악안면부의 골절에 대한 설명으로 가장 옳은 것을 한 가지만 고르시오.** | 2 |
| (Choose the most accurate statement about fractures in the oral and maxillofacial region.) |  |
| ① 골절의 방사선학적 특징 중 간접 징후로 골절선, 중첩, 계단상, 비정상적 선형 방사선불투과상, 해부학적 구조물의 소실이 관찰될 수 있다. |  |
| (Indirect signs of a fracture on radiography can include fracture lines, overlapping, stair-step patterns, abnormal linear radiolucency, or loss of anatomical structures.) |  |
| ② 하악골 골절은 하악과두 골절이 가장 흔하고 하악과두 골절 진단에는 역타운방사선영상이 유용하다. |  |
| (Mandibular fractures most commonly occur at the condyle, and reverse Towne’s radiography is useful in diagnosing condylar fractures.) |  |
| ③ 구치부 치조골 골절로 상악동의 점막 종창이나 공기-유체 수준이 생기면 이하두정촬영술로 확인할 수 있다. |  |
| (If swelling of the maxillary sinus mucosa or air-fluid levels occur from posterior alveolar bone fractures, they can be confirmed with submentovertex radiographs.) |  |
| ④ 상악골 골절 및 안면중앙부 골절은 해부학적 구조가 단순하고 구조물 중첩이 적어 일반방사선영상에서 평가하기 쉬운 편이다. |  |
| (Maxillary and midfacial fractures are easier to evaluate with standard radiography because of simpler anatomy and fewer overlapping structures.) |  |
| ⑤ 골절 치료 후 2주 정도가 지나서 골절 부위가 확장되는 경우에는 치유가 지연되고 있음을 암시하는 것이다. |  |
| (If the fracture site expands about two weeks after treatment, it could indicate delayed healing.) |  |
| **47. 구강악안면부에 발현되는 내분비장애에 대한 설명으로 가장 옳지 않은 것을 한 가지만 고르시오.** | 3 |
| (Choose the most inaccurate statement about endocrine disorders manifesting in the oral and maxillofacial region.) |  |
| ① 부갑상선저하증은 약물, 방사선, 갑상선 수술이나 자가면역에 의하여 부갑상선이 손상되었을 때 발생하며 저칼슘혈증이 나타난다. |  |
| (Hypoparathyroidism can occur due to damage to the parathyroid gland from medication, radiation, thyroid surgery, or autoimmunity, leading to hypocalcemia.) |  |
| ② 뇌하수체기능항진증은 성장호르몬이 과다하게 분비되어 과성장이 나타난다. |  |
| (Hyperfunction of the pituitary gland causes excessive secretion of growth hormone, leading to overgrowth.) |  |
| ③ 갑상선항진증은 티록신이 과다 분비되는 질환으로 치아의 발육과 유치의 탈락이 지연된다. |  |
| (Hyperthyroidism is a condition involving excessive thyroxine secretion, which can delay tooth development and primary tooth loss.) |  |
| ④ 조절되지 않는 당뇨병 환자는 치주질환 이환이 쉽고 빠르게 진행되는 경향이 있으나 방사선학적 소견은 비특이적이다. |  |
| (Patients with uncontrolled diabetes tend to develop periodontal diseases quickly, but their radiographic findings are non-specific.) |  |
| ⑤ 쿠싱병은 당질 코르티코이드가 과잉 분비되었을 때 발생하며 주된 방사선학적 소견은 골다공증이다. |  |
| (Cushing’s syndrome arises from excessive secretion of glucocorticoids, and its primary radiographic finding is osteoporosis.) |  |
| **48. 구강악안면부에 발현되는 전신질환에 대한 설명으로 가장 옳지 않은 것을 한 가지만 고르시오.** | 3 |
| (Choose the most inaccurate statement about systemic diseases manifesting in the oral and maxillofacial region.) |  |
| ① 골다공증은 폐경 이후 여성에서 흔하며 방사선학적 소견은 골밀도 감소이며 파노라마방사선영상에서 하악 하연 피질골의 비박이 관찰되기도 한다. |  |
| (Osteoporosis is common in postmenopausal women, and its characteristic radiographic finding is decreased bone density. Thinning of the mandibular lower cortex might be observed on panoramic radiographs.) |  |
| ② 구루병 및 골연화증은 비타민D 결핍으로 나타나며 구루병은 치아의 발육과 맹출 지연이 나타나나 골연화증은 치아 자체에는 영향을 미치지 않는다. |  |
| (Rickets and osteomalacia result from vitamin D deficiency. Rickets can cause delayed tooth development and eruption, while osteomalacia does not affect the teeth.) |  |
| ③ 저인산증의 방사선학적 소견은 전반적 방사선투과성 증가, 피질골과 치조백선 비박이 관찰되며 치아에서는 치수강과 근관 폐쇄가 관찰된다. |  |
| (The radiographic findings of hypophosphatasia include generalized increased radiolucency, thinning of cortical bone, and loss of the lamina dura. Teeth might show closed pulp chambers and root canals.) |  |
| ④ 신장성 골형성장애의 방사선학적 소견은 악골에서 치조백선의 소실을 보이며 하악각 부위에서는 피질골의 두께가 감소한다. |  |
| (The radiographic findings of renal osteodystrophy include loss of the lamina dura in the jaw and reduced cortical bone thickness in the mandibular angle area.) |  |
| ⑤ 진행전신경화증은 치주인대강의 확장이 환자의 약 2/3에서 관찰되며 초기 골육종과 감별진단해야 한다. |  |
| (Progressive systemic sclerosis can present as periodontal ligament widening in about two-thirds of patients, which should be distinguished from early signs of osteosarcoma.) |  |
| **49. 연조직석회화에 대한 설명으로 가장 옳은 것을 한 가지만 고르시오.** | 5 |
| (Choose the most accurate statement about soft tissue calcification.) |  |
| ① 정맥석은 하악관 하방의 설골과 경추 사이에 방사선불투과상으로 나타난다. |  |
| (Phleboliths appear as radiopaque masses between the hyoid bone below the mandibular canal and cervical vertebrae.) |  |
| ② 동맥석회화는 원형 또는 타원형의 균일한 방사선불투과성으로 나타나며 간혹 특징적인 동심원의 석회침착으로 보이기도 한다. |  |
| (Arterial calcifications can present as uniform radiopaque circles or ovals, and sometimes as concentric rings of calcification.) |  |
| ③ 상악동석은 크기, 밀도, 형태가 다양하게 나타나며 상악동 내로 들어간 치근파절편과 감별하여야 하며 수술로 제거해야 한다. |  |
| (Antroliths vary in size, density, and shape. They need to be differentiated from root fragments intruding into the sinus, and surgical removal may be required.) |  |
| ④ 경상설골인대의 골화는 파노라마 영상에서 주로 편측성으로 나타나고 대개 직선형이나 간혹 분절된 양상을 보이기도 한다. |  |
| (Ossification of the stylohyoid ligament is generally unilateral on panoramic radiographs, often appearing as a straight line, but it may also be segmented.) |  |
| ⑤ 피부골종이 치근과 중첩되어 나타나면 골경화증과 감별이 어려운 경우도 있는데 뺨을 부풀린 상태에서 방사선영상을 획득하면 연조직 내의 병소 위치를 확인할 수 있다. |  |
| (If osteoma cutis overlaps with roots, it can be challenging to distinguish this condition from osteosclerosis. A puffed-cheek radiograph can help confirm the location of the lesion within the soft tissue.) |  |
| **50. 구강악안면부 발육장애에 대한 설명으로 가장 옳지 않은 것을 한 가지만 고르시오.** | 2 |
| (Choose the most inaccurate statement regarding oral and maxillofacial development disorders.) |  |
| ① 두개안면이골증은 두부방사선영상에서 두개봉합이 관찰되지 않고 손가락 자국, 쇠를 두드린 모양이라고도 하는 두개흔이 관찰된다. |  |
| (Craniofacial dysostosis does not show cranial sutures on cephalometric radiographs, and cranial markings that look like finger marks or beaten metal are observed.) |  |
| ② 하악안면이골증은 관골이나 상악동의 발육 부전을 보이며 하악전돌증의 특징을 보인다. |  |
| (Mandibulofacial dysostosis shows underdevelopment of the zygomatic bone or maxillary sinus, and features of mandibular prognathism.) |  |
| ③ 구개열은 치조골의 파열 부위에서 치아의 이상 소견이 자주 관찰되어 상악 측절치의 결손이 관찰되며 종종 치아의 형성장애, 위치 이상이 나타나기도 한다. |  |
| (Cleft palate often involves tooth abnormalities at the site of the alveolar cleft, with frequent observations of missing maxillary lateral incisors, as well as tooth malformations or malposition in some cases.) |  |
| ④ 근돌기 과다형성은 파노라마방사선영상이나 워터스방사선영상에서 비대해진 근돌기가 관찰되며 최대 개구 상태에서 촬영한 CT 영상에서 근돌기와 관골돌기 후벽의 근접 정도를 확인할 수 있다. |  |
| (Hyperplasia of the coronoid process is identified on panoramic radiographs or Waters radiographs showing an enlarged coronoid process, and its proximity to the zygomatic arch’s posterior wall can be verified in CT images taken with maximum mouth opening.) |  |
| ⑤ 편측안면과다형성은 이환측 상, 하악과 관골의 비대 및 치열의 이상이 관찰되며 편측안면형성저하와 감별이 필요하다. |  |
| (Hemifacial hyperplasia displays hypertrophy of the affected side’s maxilla, mandible, and zygomatic bone. It must be distinguished from hemifacial hypoplasia in the differential diagnosis.) |  |
| **51. 측두하악관절의 영상진단에 대한 설명으로 가장 옳지 않은 것을 한 가지만 고르시오.** | 3 |
| (Choose the most inaccurate statement regarding imaging diagnoses of the temporomandibular joint.) |  |
| ① MRI는 관절원반의 위치, 형태 등을 관찰할 수 있고 T1 강조 영상이나 PD 영상에서는 관절원반의 위치와 모양을 평가하고 T2 강조 영상에서는 염증과 삼출을 평가한다. |  |
| (MRI can be used to observe the position and shape of the articular disc; T1-weighted or PD images are used to assess the position of the articular disc, and T2-weighted images can evaluate inflammation and effusion.) |  |
| ② 하악골이 성장하는 동안에 하악과두 골절이 발생하면 하악과두의 저형성 가능성이 있다. |  |
| (If a mandibular condyle fracture occurs during mandibular growth, there is a possibility of underdevelopment of the mandibular condyle.) |  |
| ③ 하악과두 골절의 약 60%에서 골편의 변위가 관찰되며 일반적으로 내측익돌근의 수축에 의해 골절된 골편은 전내방으로 변위된다. |  |
| (About 60% of mandibular condyle fractures show displacement of the fractured condylar fragment, and it is typically displaced anteriorly and medially due to the contraction of the medial pterygoid muscle.) |  |
| ④ 측두하악관절 강직의 진성 강직 중 섬유성 강직은 이전의 관절염이나 외상의 조직 치유 과정에서 발생하는 섬유성 유착에 의한 개구장애를 말한다. |  |
| (Fibrous ankylosis, within the category of true ankylosis of temporomandibular joint, refers to a mouth-opening limitation due to fibrous adhesions occurring during tissue healing after previous arthritis or trauma.) |  |
| ⑤ 측두하악에 발생하는 악성종양으로는 연골육종, 골육종, 활액육종 등이 있으며 극히 드물게 발생하고 유방, 신장, 폐 등에 발생한 악성종양이 전이되는 경우도 있다. |  |
| (Malignant tumors of the temporomandibular joint include chondrosarcoma, osteosarcoma, and synovial sarcoma. They occur extremely rarely, and metastases from malignancies in other areas such as the breast, kidney, or lung may be observed.) |  |
| - **Image interpretation (n=9)** |  |

| - **Short-answer question (n=1)** |  |
| --- | --- |

| **52. 제시글의 빈 칸에 알맞은 용어를 쓰시오.** | 이하두정방사선영상  (submentovertex radiograph) |
| --- | --- |
| (Fill in the blank with the correct term.) |  |
| 구외방사선촬영 중 관골 골절 여부를 확인하려면 X선 노출을 줄여 촬영한 ( )이 유용하다. |  |
| (Among extraoral radiographs, ( ), taken with reduced radiation exposure, is useful for detecting zygomatic bone fractures.) |  |
